# Single-cell RNA sequencing analysis reveals the critical role of fibroblasts in aortic progeria-associated vascular remodeling in Hutchinson-Gilford progeria syndrome mice

**DOI:** 10.3389/fimmu.2025.1638083

**Published:** 2025-10-24

**Authors:** Qian Sun, Liehao Yang, Zilong Zhou, Nan Wu, Chunyi Li, Qianying Hu, Xianling Cong

**Affiliations:** ^1^ Department of Dermatology, China-Japan Union Hospital of Jilin University, Changchun, China; ^2^ Department of Biobank, China-Japan Union Hospital of Jilin University, Changchun, China; ^3^ Phase I Clinical Trial Research Laboratory, China-Japan Union Hospital of Jilin University, Changchun, China; ^4^ Institute of Antler Science and Product Technology, Changchun Sci-Tech University, Changchun, China

**Keywords:** HGPS, fibroblasts, single-cell transcriptome analysis, aorta, aging

## Abstract

**Background:**

Patients with Hutchinson-Gilford progeria syndrome (HGPS) typically succumb to cardiovascular diseases in their teens. Although fibroblasts have been implicated in the progression of arteriosclerosis, their roles and mechanisms in progeroid aorta remain poorly understood.

**Methods:**

Utilizing single-cell RNA sequencing, we analyzed aortic tissues from HGPS mice with a focus on fibroblasts. Through gene expression profiling, Gene Ontology (GO) analysis, and cell-cell communication networks across various cell types, we revealed the unique contributions of fibroblasts during HGPS aortic aging. Finally, knockdown of *Lgals3bp* in HGPS cells was performed to investigate its role in inflammation and fibrosis.

**Results:**

Fibroblasts exhibited altered gene expression profiles associated with extracellular matrix dysregulation and inflammatory modulation, along with elevated senescence-associated secretory phenotype (SASP) scores in HGPS mice. Fibroblasts demonstrated the highest interaction frequency and intensity among aortic cell populations, with the strongest intercellular crosstalk observed between fibroblasts and dysfunctional vascular smooth muscle cells. We defined nine fibroblast subclusters and delineated their distinct transcriptional signatures, developmental trajectories, and interaction networks. Additionally, we identified significant upregulation of Lgals3bp in aortic fibroblasts of HGPS mice, which promoted the expression of pro-inflammatory factors and fibrosis-related genes.

**Conclusion:**

Our findings underscore the pivotal role of fibroblasts in aortic progeria-associated vascular remodeling in HGPS mice and suggest that *Lgals3bp* may represent a potential therapeutic target for aortic pathology in HGPS.

## 1 Introduction

Hutchinson-Gilford progeria syndrome (HGPS) is a rare genetic disorder caused by LMNA gene mutations and aberrant expression of progerin. It is characterized by accelerated aging and premature mortality, and most patients succumb to cardiovascular diseases in their teenage years (1–3). The aortic pathology in HGPS is marked by increased vascular fibrosis, extracellular matrix (ECM) accumulation, and loss of vascular smooth muscle cells (VSMCs), which ultimately drive atherosclerosis (4, 5). These vascular phenotypes likely arise from dysregulated cellular composition, molecular alterations, and disrupted intercellular signaling within the vascular wall. Although recent studies have predominantly focused on endothelial cells (ECs) and VSMCs dysfunction in HGPS (6–8), the roles and underlying mechanisms of fibroblasts in this process remain poorly understood.

Fibroblasts are ECM-producing cells in the vasculature and contribute to atherosclerosis (9). Impaired lysosomal activity and autophagy in fibroblasts, along with the induction of apoptosis, can accelerate the progression of atherosclerosis (10). Recent studies have shown that in advanced human carotid atherosclerosis, fibroblasts promote plaque progression by activating complement and coagulation pathways in macrophages (11). These findings underscore the important role fibroblasts play in vascular remodeling of the aorta. Therefore, it is important to characterize fibroblast alterations in the arteries of HGPS mice and their impact on other vascular cell types to understand disease pathogenesis.

Single-cell RNA sequencing (scRNA-seq) of aged vasculature enables the mapping of diverse cellular landscapes and the identification of cell type-specific regulatory changes during senescence (12, 13). Previous scRNA-seq analyses of HGPS mouse aortas have primarily focused on the potential impact of ECs on HGPS vascular pathology, with limited attention being paid to the role of fibroblasts (6). In this study, we reanalyzed these sequencing datasets with a focus on the role of fibroblasts, aiming to investigate how fibroblasts influence the aortic remodeling of HGPS mice and to seek a promising target for ameliorating aortic inflammation and fibrosis. Collectively, our findings aim to provide a deeper understanding of the functions and mechanisms of fibroblasts in progeroid aorta.

## 2 Results

### 2.1 Aortic aging-associated vascular remodeling in *Lmna^G609G/G609G^* mice

**Figure 1:**
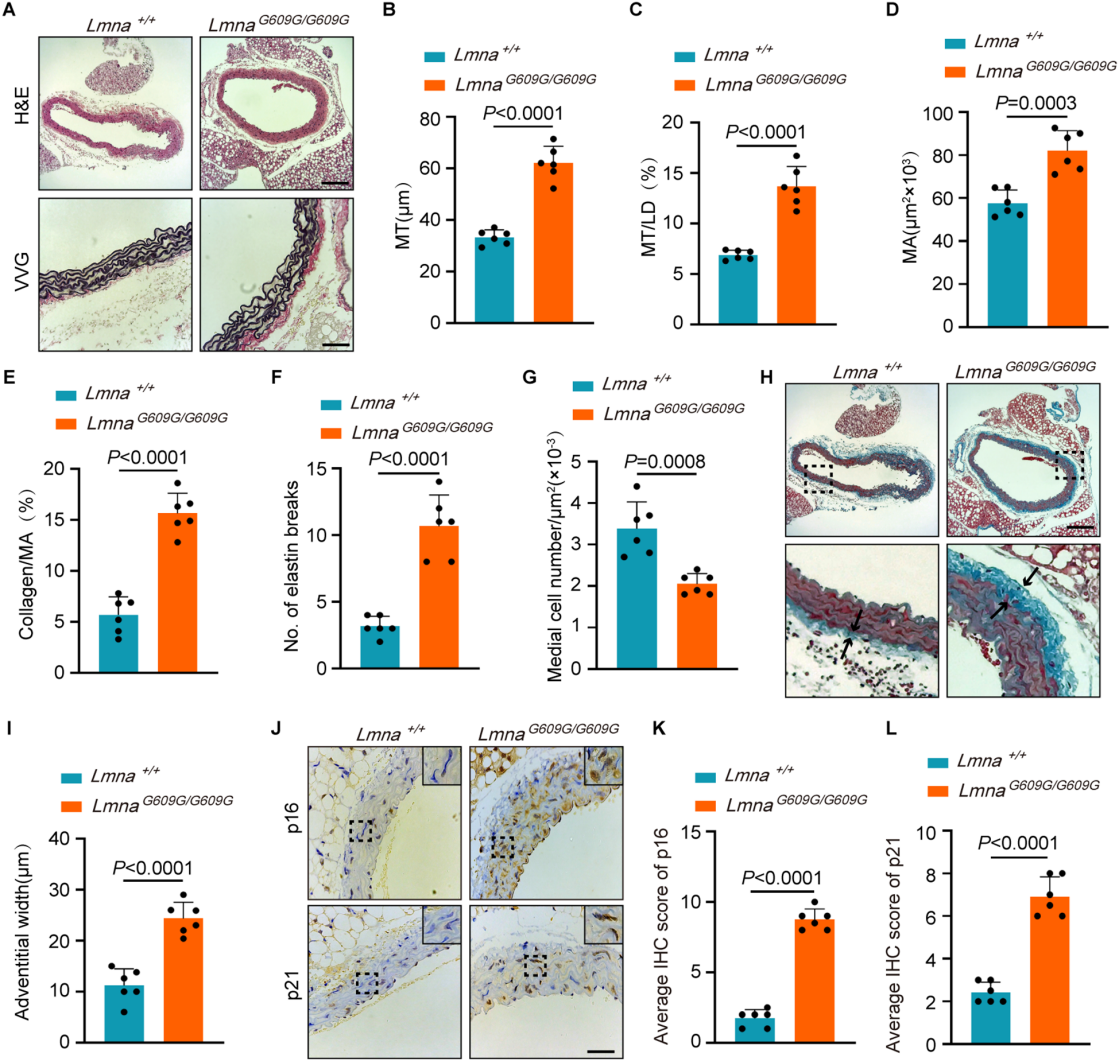
Aortic aging-associated vascular remodeling in *Lmna^G609G/G609G^* mice. **(A-G)** Hematoxylin-eosin (H&E) and Verhoeff-van Gieson (VVG) staining of *Lmna^+/+^* and *Lmna^G609G/G609G^* mouse aorta (14 weeks). Scale bar, 150 μm (H&E); 50 μm (VVG) **(A)**. Quantification of media thickness (MT) **(B)**, MT/lumen diameter (LD) **(C)**, media area (MA) **(D)**, collagen/MA **(E)**, elastin breaks **(F)**, and medial cell number **(G)** (n=6). **(H, I)** Masson’s trichrome staining of the aortic adventitial fibrosis (blue areas) from *Lmna^+/+^* and *Lmna^G609G/G609G^* mice (14 weeks). Scale bar, 150 μm. The boxes and arrows denoted the aortic adventitia **(H)**. Quantification of aortic adventitial thickness (n=6) **(I)**. **(J-L)** Immunohistochemistry (IHC) of p16 and p21 in the aorta of *Lmna^+/+^* and *Lmna^G609G/G609G^* mice. Scale bar, 50 μm **(J)**. The quantification scores of positive signals (n=6) **(K, L)**. Data are presented as mean ± SD. Statistical significance was determined by two-tailed unpaired Student’s t-test.

To assess aortic aging in *Lmna^G609G/G609G^* mice, we first evaluated morphological changes using hematoxylin and eosin (H&E) and Verhoeff–Van Gieson (VVG) staining. Compared to *Lmna^+/+^* littermates, *Lmna^G609G/G609G^* mice exhibited hallmarks of vascular aging, including increased media thickness, elevated media-to-lumen diameter ratio, expanded media area, higher collagen-to-media area ratio, elastin disruption, and reduced medial cell density (**Figures 1A-G**). Masson’s trichrome staining revealed thickened aortic adventitia in *Lmna^G609G/G609G^* mice (**Figures 1H, I**). Additionally, immunohistochemistry demonstrated significantly upregulated expression of the senescence markers, p16 and p21, in aortas of *Lmna^G609G/G609G^* mice (**Figures 1J-L**). Collectively, these findings indicate that *Lmna^G609G/G609G^* mice develop aging-associated vascular remodeling in the aorta.

### 2.2 Single-cell RNA sequencing reveals distinct cell clusters and expression patterns in aortas of *Lmna* ^+/+^ and *Lmna* ^G609G/G609G^ mice

**Figure 2:**
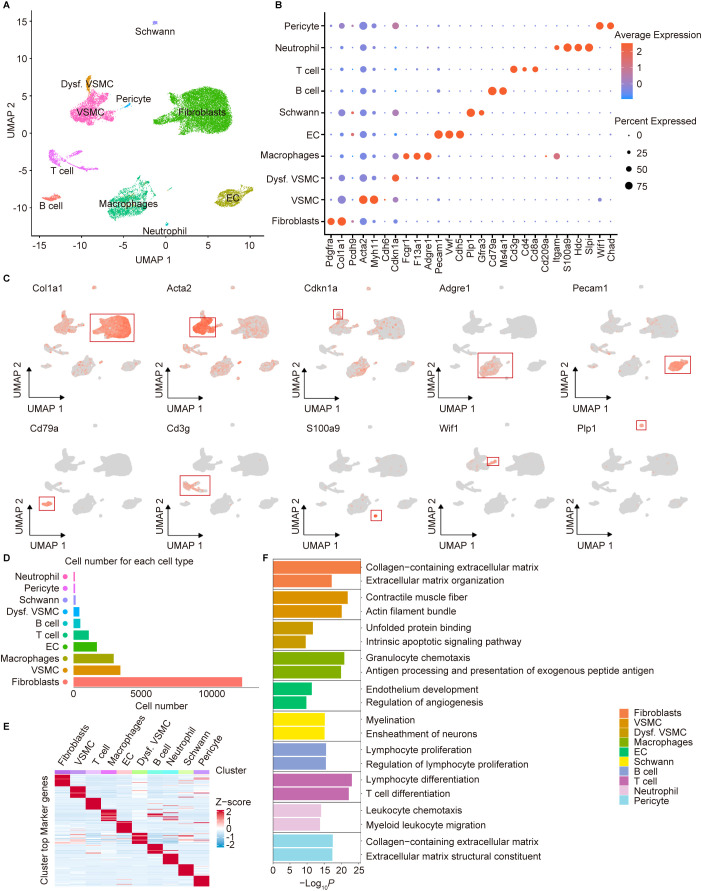
Cell types identified by scRNA-seq of aortic tissues in *Lmna^+/+^* and *Lmna^G609G/G609G^* mice. **(A)** Uniform manifold approximation and projection (UMAP) plot showing the ten cell types of mouse aorta. VSMC, vascular smooth muscle cell; Dysf. VSMC, dysfunctional VSMC; EC, endothelial cell. **(B)** Dot plot showing the expression of representative genes for each cell type. **(C)** UMAP plots showing the representative genes of each cell type. The color key from gray to red indicates low to high gene expression levels. **(D)** Bar chart showing the cell number of each cell type. **(E)** Heatmap showing the gene expression signatures of each cell type. **(F)** Gene Ontology (GO) enrichment analysis for each cell type.

To investigate cell type-specific transcriptional alterations in the aortas of *Lmna^G609G/G609G^* mice at single-cell resolution, we reanalyzed scRNA-seq datasets derived from the aortic tissues of 14-week-old *Lmna^G609G/G609G^* and *Lmna^+/+^* mice (6). Uniform manifold approximation and projection (UMAP) dimensionality reduction were employed to visualize global aortic cell populations and 10 distinct cell types, based on their expression of canonical markers, were identified: fibroblasts, VSMCs, dysfunctional VSMCs, macrophages, ECs, Schwann cells, B cells, T cells, neutrophils, and pericytes (**Figure 2A**). Dot and UMAP plots illustrated marker genes defining each cell type (**Figures 2B, C**). To quantify cellular composition, we generated a bar plot depicting the proportional abundance of each cell population in the aortas (**Figure 2D**). Fibroblasts constituted the predominant population in the aortic cellular landscape.

GO analysis of the top 20 marker genes for each cell type revealed the biological functions and features corresponding to the known characteristics of each cell cluster (**Figures 2E, F**). For example, GO terms such as “collagen-containing extracellular matrix” and “extracellular matrix organization” aligned with the molecular signatures of fibroblasts, while “contractile muscle fiber” and “actin filament bundle” corresponded to those of VSMCs. Similarly, “endothelium development” and “regulation of angiogenesis” reflected the functional profiles of ECs. Additionally, immune-related terms, including “granulocyte chemotaxis”, “lymphocyte proliferation”, “lymphocyte differentiation”, and “leukocyte chemotaxis”, were associated with macrophages, B cells, T cells, and neutrophils, respectively. In summary, our results define the molecular signatures and functional heterogeneity of distinct cell populations in the aortas of *Lmna^+/+^* and *Lmna^G609G/G609G^* mice.

### 2.3 Aortic cell type-specific transcriptomic alterations in *Lmna^G609G/G609G^* mice

**Figure 3:**
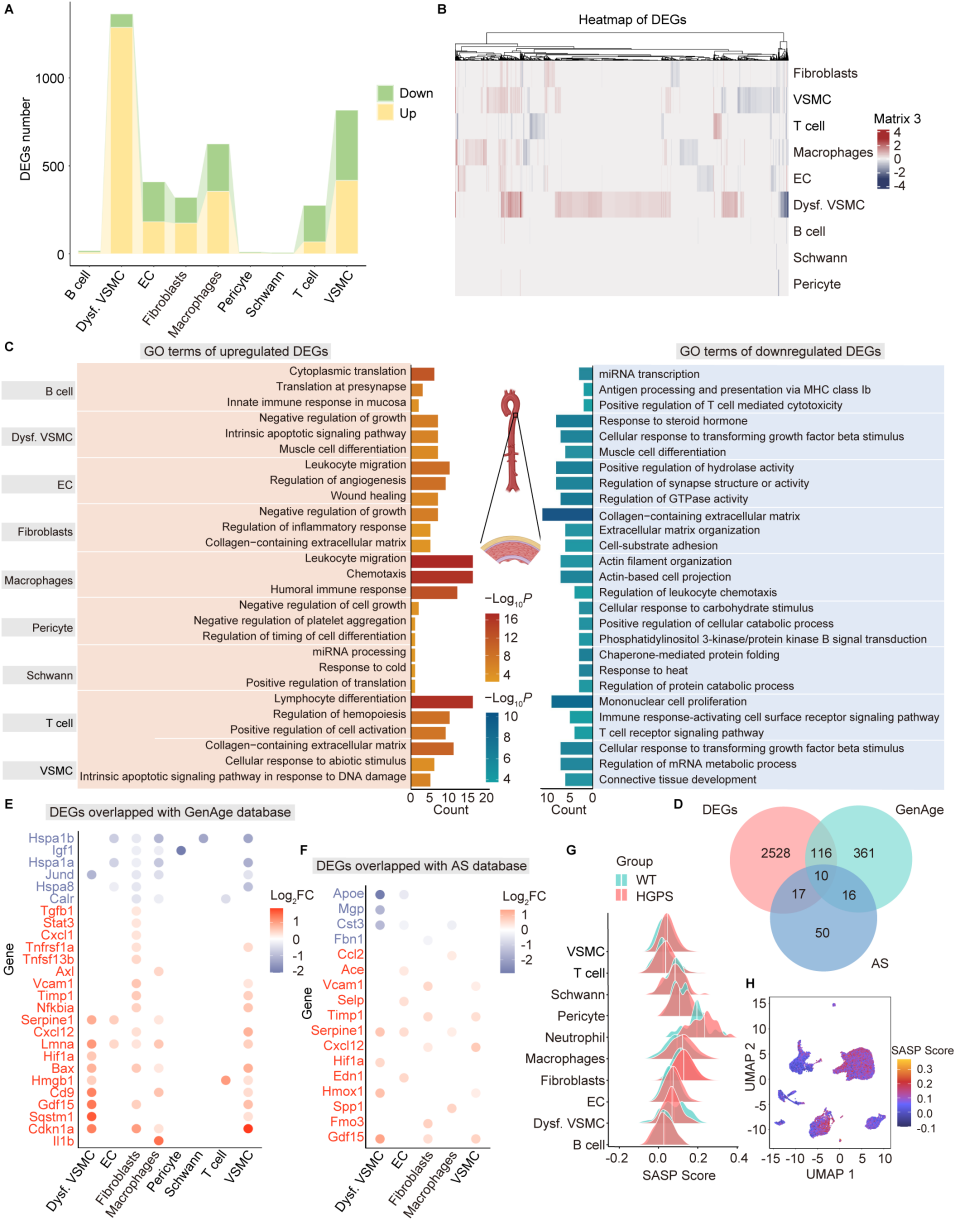
Transcriptional alterations in various cell types of aortic tissues in *Lmna^G609G/G609G^* mice. **(A)** Bar plot showing the numbers of differentially expressed genes (DEGs) across different cell types in the aorta of *Lmna^G609G/G609G^* mice. **(B)** Heatmap showing the DEGs across different cell types in the aorta of *Lmna^G609G/G609G^* mice. **(C)** Diagram showing the representative enrichment of GO terms of ten cell types in the aorta of *Lmna^G609G/G609G^* mice. **(D)** Venn diagram showing overlap among DEGs in the aorta of *Lmna^G609G/G609G^* mice, aging-related genes from GenAge database (https://genomics.senescence.info/genes/), and arteriosclerosis-associated genes from MalaCards human disease database (https://www.malacards.org). AS, arteriosclerosis. **(E, F)** Dot plot showing the DEGs overlapping with the GenAge database **(E)** and MalaCards arteriosclerosis database **(F)** in the aorta of *Lmna^G609G/G609G^* mice. **(G)** Ridge plot showing the shift of SASP gene set score across different cell types in the aorta of *Lmna^G609G/G609G^* mice. **(H)** UMAP plot showing the SASP score of each cell type in the aorta of *Lmna^G609G/G609G^* mice. The color key from blue to red indicates low to high SASP score.

To further explore the mechanisms underlying aortic aging at the cellular level, we compared gene expression patterns across aortic cell types between *Lmna^+/+^* and *Lmna^G609G/G609G^* mice. We identified thousands of differentially expressed genes (DEGs) in the aortas of *Lmna^G609G/G609G^* mice, including 2496 upregulated and 1351 downregulated DEGs (**Supplementary Table S1**). The most affected cell types included dysfunctional VSMCs, VSMCs, macrophages, ECs, and fibroblasts, with 1364, 817, 625, 409, and 321 DEGs, respectively (**Figures 3A, B**). GO enrichment analysis revealed that upregulated genes were predominantly associated with leukocyte migration and chemotaxis, whereas the downregulated genes were enriched in collagen-containing extracellular matrix and mononuclear cell proliferation (**Figure 3C**), reflecting chronic inflammation and disrupted ECM homeostasis in HGPS aortas.

To further explore the molecular links between aortic aging and atherosclerosis in HGPS, we performed a comprehensive comparative analysis of DEGs with age-related genes from the GenAge database and arteriosclerosis-related genes from the MalaCards Human Disease database (**Figure 3D**). We identified 126 age-related genes with differential expression in HGPS aortas, such as *Cdkn1a*, *Stat3*, *Lmna*, and *Il1b* (**Figure 3E**). Concurrently, 27 DEGs were hot spots related to atherosclerosis, such as *Ace*, *Apoe*, and *Hif1a* (**Figure 3F**). These findings probably imply a transcriptional convergence between HGPS vascular aging and arteriosclerosis. Strikingly, 10 genes exhibited shared dysregulation in both aging and atherosclerosis, including *Vcam1*, *Apoe*, *Gdf15*, *Timp1*, *Cxcl12*, *Hspd1*, *Serpine1*, *App*, *Eln*, and *Hif1a* (**Figure 3D**), suggesting their potentially critical roles in the atherosclerosis driven by aging in HGPS mice.

To further evaluate age-related inflammation of different cell types in the aorta of *Lmna^G609G/G609G^* mice, we performed senescence-associated secretory phenotype (SASP) scoring across distinct cell populations. Our analysis revealed significant increase in the SASP score across six aortic cell types in *Lmna^G609G/G609G^* mice, including ECs, fibroblasts, VSMCs, macrophages, neutrophils, and T cells (**Figure 3G**), indicating that the aorta of HGPS mice is characterized by chronic inflammation. Notably, fibroblasts and macrophages exhibited the highest SASP scores, suggesting their predominant role in driving chronic inflammation during HGPS aortic aging.

### 2.4 Enhanced fibroblast communication with dysfunctional VSMCs during aortic aging in HGPS mice

**Figure 4:**
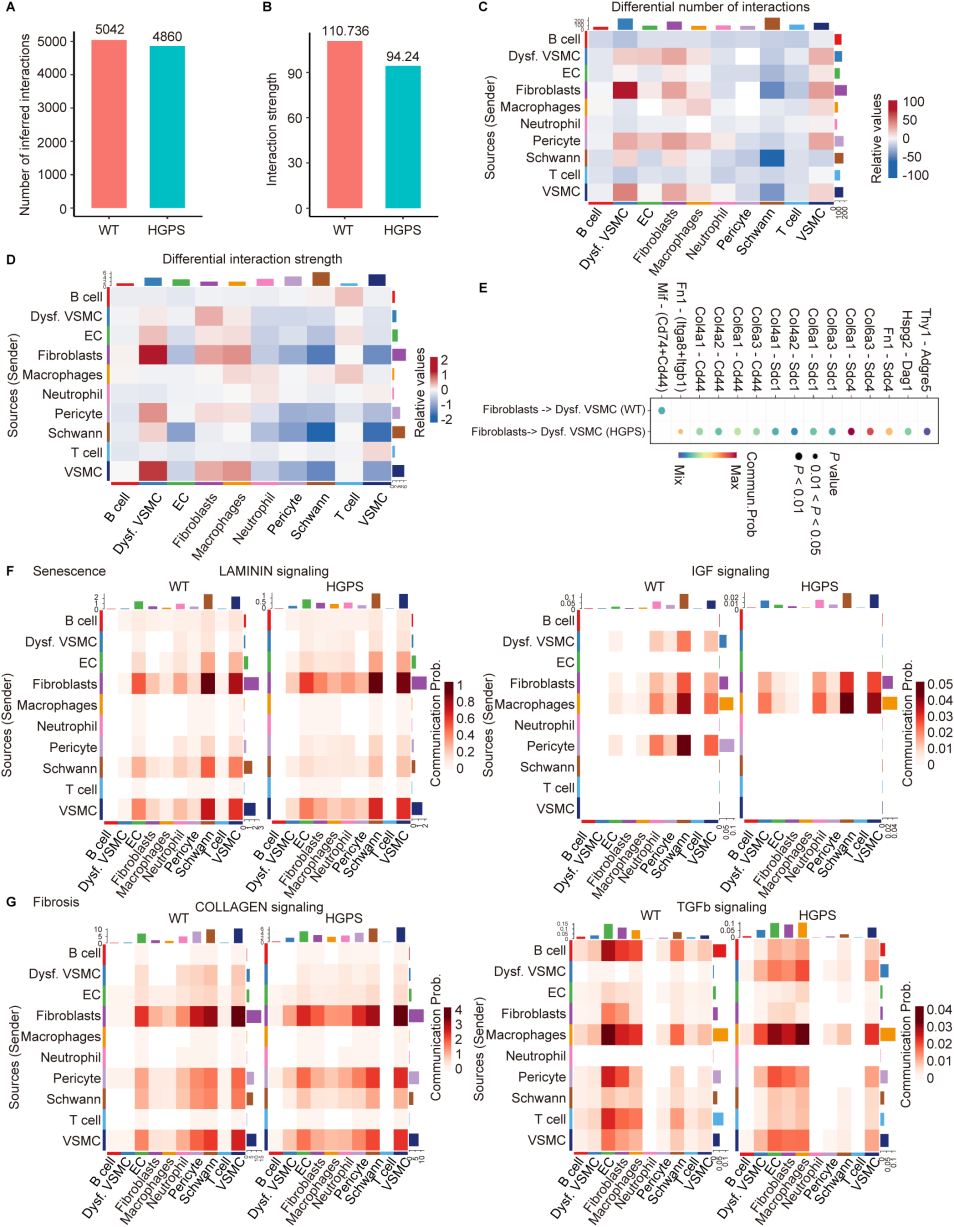
Cell-cell communication in the aorta of *Lmna^+/+^* and *Lmna^G609G/G609G^* mice. **(A, B)** Bar plots represent the changes in the total number of cell interactions **(A)** and the overall interaction strength **(B)** in the aorta of *Lmna^+/+^* and *Lmna^G609G/G609G^* mice. **(C, D)** Heatmaps showing the differential number **(C)** and the strength **(D)** of cell-cell communication in the aorta of *Lmna^G609G/G609G^* mice. The x axis represents target (receiver) cell types, and the y axis represents source (sender) cell types. **(E)** Communication strength of all significant signalling pathways from fibroblasts to dysfunctional VSMCs in the aorta of *Lmna^+/+^* and *Lmna^G609G/G609G^* mice. **(F)** Heatmaps showing the senescence phenotype is explored through LAMININ and IGF signaling pathways in the aorta of *Lmna^+/+^* and *Lmna^G609G/G609G^* mice. **(G)** Heatmaps showing the fibrosis phenotype is explored through COLLAGEN and TGFb signaling pathways in the aorta of *Lmna^+/+^* and *Lmna^G609G/G609G^* mice.

Given that aging is thought to affect intercellular communication, we employed the R package “CellChat” to explore communication and interactions among the 10 identified cell populations. Compared with wild-type (WT) controls, we observed a reduction in the overall frequency and strength of cellular interactions in HGPS aortas (**Figures 4A, B**). Fibroblasts exhibited the highest frequency and intensity of signaling. In particular, in the aorta of HGPS mice, the number and strength of communication from fibroblasts to dysfunctional VSMCs were markedly increased (**Figures 4C, D**). Ligand-receptor pairs, including COL family syndecan receptors and FN1-integrin receptors, played crucial roles in mediating interactions between fibroblasts and dysfunctional VSMCs in the aorta of HGPS mice (**Figure 4E**). This suggests that fibroblasts may affect the function of VSMCs through the secretion of collagen and FN1. Furthermore, pathways associated with senescence (e.g., laminin, IGF) and fibrosis (e.g., COLLAGEN) were upregulated in fibroblast-to-dysfunctional VSMC communication in HGPS aortas, while the TGF-β signaling pathway was markedly activated in dysfunctional VSMCs (**Figures 4F, G**). These findings suggest that fibroblasts may play an important role in mediating VSMC dysfunction during accelerated aging of the aorta in HGPS mice; however, this hypothesis requires future experimental validation.

### 2.5 Functional annotation and developmental trajectories of fibroblast subpopulations

**Figure 5:**
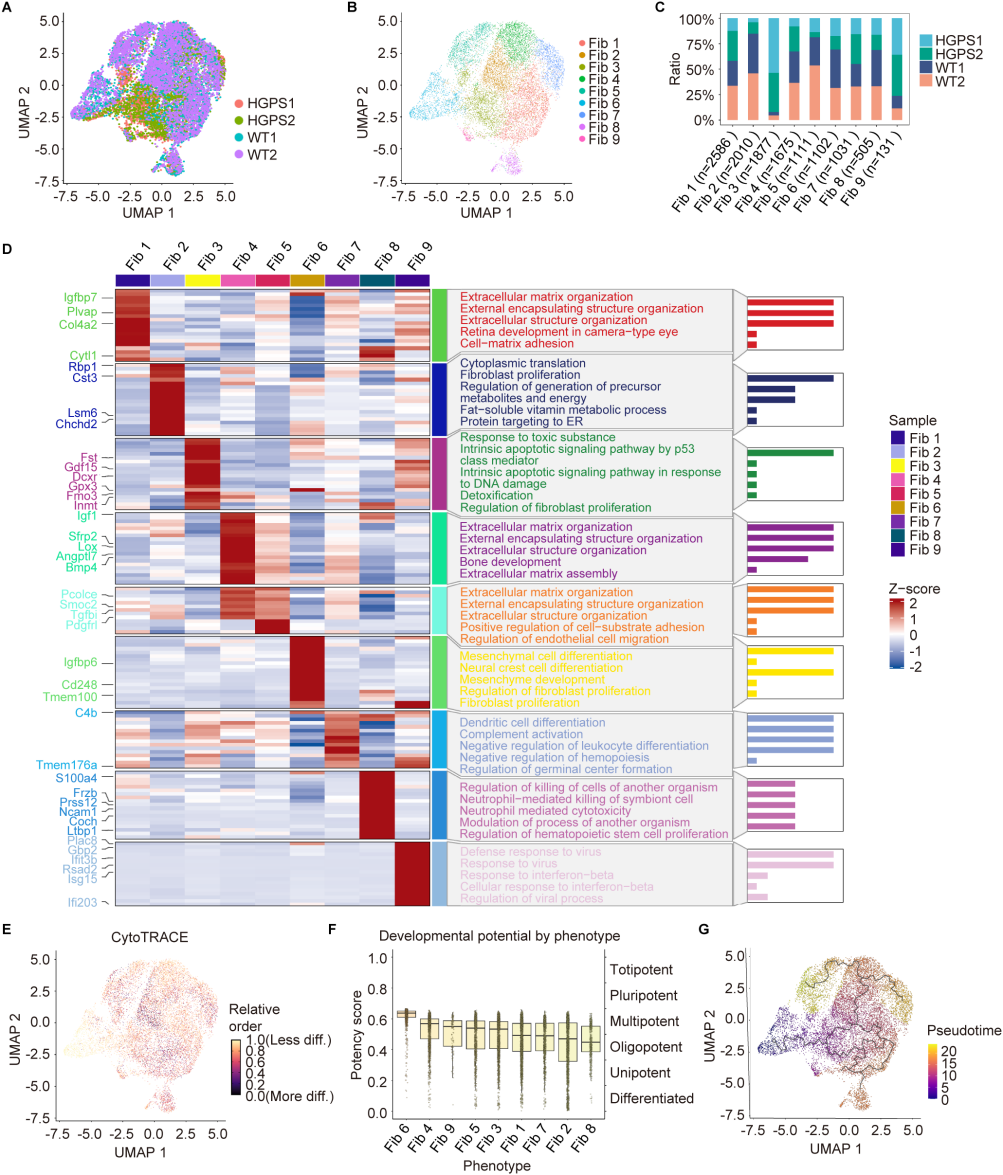
Functional annotation and pseudotime trajectory analysis of fibroblast subpopulations. **(A)** UMAP plot showing the distribution of aortic fibroblasts from WT and HGPS mice. **(B)** UMAP plot showing the distinct fibroblast subpopulations in the mouse aorta. **(C)** The proportion of fibroblast subpopulations in the aorta of WT and HGPS mice. **(D)** Heatmap showing the top 5 enriched entries for GO enrichment analysis of DEGs among fibroblast subpopulations. **(E)** CytoTRACE analysis among fibroblast subpopulations. **(F)** Boxplots showing CytoTRACE research of the developmental potential of fibroblast subpopulations. **(G)** UMAP plot showing the distribution of differentiation trajectories of fibroblasts in fibroblast subpopulations. Solid lines indicated differentiation trajectories.

Given our observation that fibroblasts played a critical role in aging-associated vascular remodeling in the aortas of HGPS mice, and considering their inherent heterogeneity and plasticity, we performed subclustering of fibroblasts to uncover the distinct roles of fibroblast subclusters in progeroid aorta. Using UMAP-based clustering, we segregated all fibroblasts into nine subclusters and observed a significant increase in the proportion of HGPS cells in Fib3 and Fib9, while the most significant increase in WT cells was observed in Fib2, Fib5, and Fib6 (**Figures 5A-C**).

To gain deeper insight into the functional characteristics of the nine fibroblast subclusters, we performed GO enrichment analysis on highly variable genes. Fib3 was associated with the response to toxic substances, p53-mediated intrinsic apoptosis, and DNA damage response pathways, implying a redox imbalance and a propensity for cell cycle arrest or apoptosis. Fib9 was enriched in antiviral defense and interferon-β response pathways, highlighting its role in interferon signaling and antiviral responses, thereby promoting inflammatory reactions (**Figure 5D**). The increased proportions of Fib3 and Fib9 in HGPS aortas reflect an accumulation of senescent cells accompanied by heightened inflammatory activity. Fib2 was enriched in cytoplasmic translation, fibroblast proliferation, and regulation of precursor metabolite and energy generation, indicative of a proliferative and metabolically active state. Fib5 was linked to ECM organization, positive regulation of cell-substrate adhesion, and regulation of extracellular matrix endothelial cell migration, suggesting a dual role in ECM synthesis and endothelial cell migration. Fib6 was enriched in mesenchymal differentiation and fibroblast proliferation, indicating its potential differentiation capacity (**Figure 5D**). The reduced abundance of Fib2, Fib5, and Fib6 in HGPS aortas suggests the disruption of their normal functions, including proliferation, metabolism, differentiation, ECM synthesis, and endothelial migration.

To investigate the differentiation states and developmental trajectories of the fibroblast subclusters, we employed CytoTRACE and Monocle 3 pseudotemporal trajectory analyses of the nine fibroblast subpopulations. CytoTRACE analysis revealed that Fib6 exhibited the highest CytoTRACE score among all subclusters, indicating a less differentiated state (**Figures 5E, F**). Conversely, Fib8 fibroblasts had the lowest CytoTRACE score, suggesting terminal differentiation with minimal differentiation potential (**Figure 5F**). Pseudotemporal trajectory analysis positioned Fib6 as the developmental origin of fibroblast subclusters, with Fib5, Fib7, Fib2, Fib1, Fib9, and Fib8 sequentially localized toward the endpoints of the trajectory (**Figure 5G**), reflecting progressive differentiation.

### 2.6 SASP score and intercellular communication in aortic fibroblast subpopulations of *Lmna^G609G/G609G^* mice

**Figure 6:**
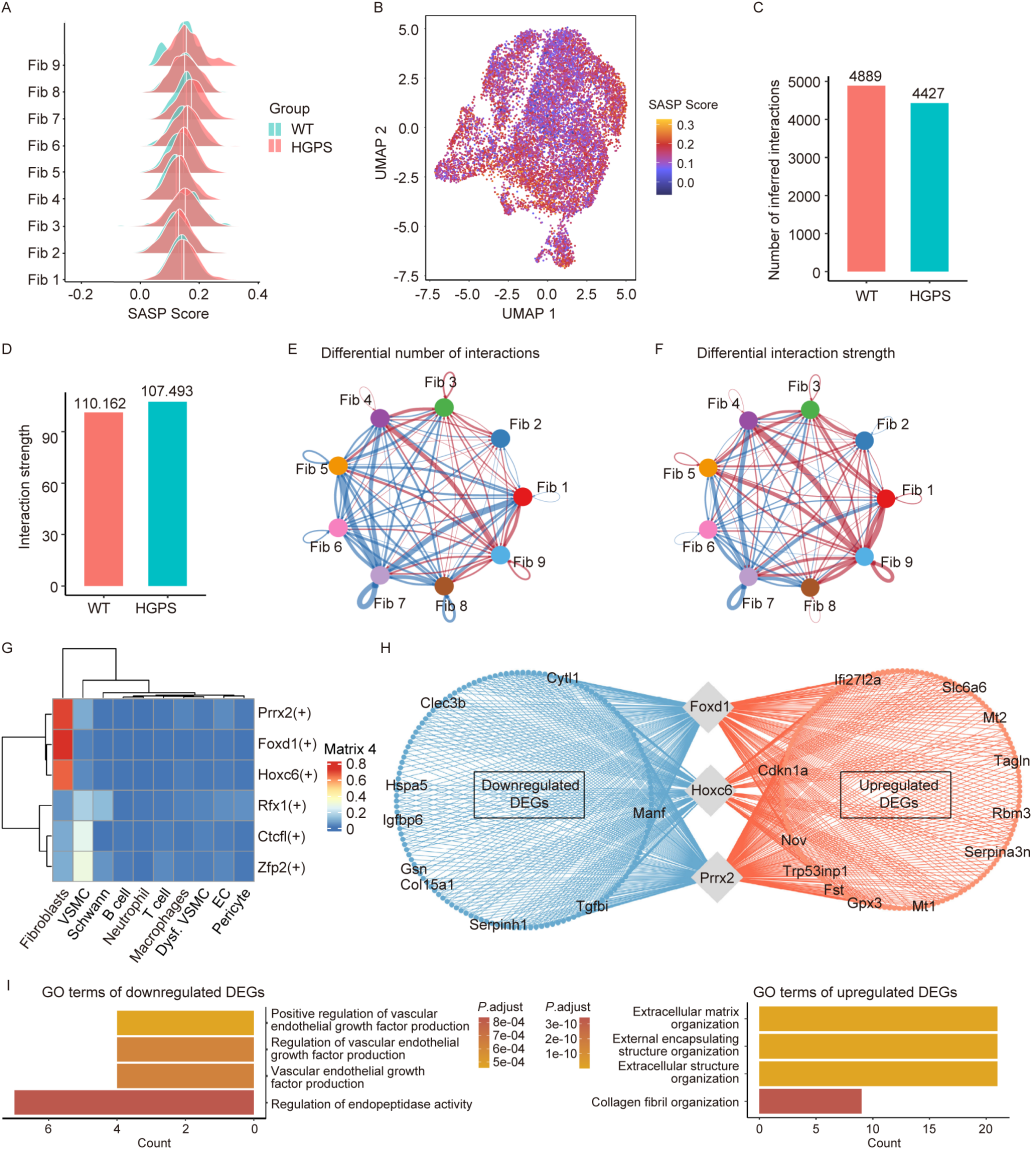
SASP score, intercellular communication, and transcriptional network analysis in Fibroblast Subpopulations. **(A)** Ridge plot showing the changes of SASP gene set scores across different fibroblast subpopulations in the aorta of *Lmna^G609G/G609G^* mice. **(B)** UMAP plot showing the SASP score of each fibroblast subpopulation in the aorta of *Lmna^G609G/G609G^* mice. The color key from blue to red indicates low to high SASP score. **(C, D)** Bar plots represent the changes in the total number of interactions **(C)** and interaction strength **(D)** between fibroblast subpopulation cells in the aorta of *Lmna^+/+^* and *Lmna^G609G/G609G^* mice. **(E, F)** Circle plots visualizing changes in the number of interactions **(E)** and interaction strength **(F)** between different fibroblast subpopulations in the aorta of *Lmna^G609G/G609G^* mice. Red lines denote enhanced communication, whereas blue lines indicate reduced interactions. Line thickness corresponds to the magnitude of change, with thicker lines representing more pronounced alterations. **(G)** Matrix plot identifies key transcription factors specifically regulating DEGs in fibroblasts. **(H)** Network plot showing the differentially expressed *Foxd1*, *Hoxc6*, and *Prrx2* target genes in fibroblasts. **(I)** GO enrichment analysis of downregulated or upregulated *Foxd1*, *Hoxc6*, and *Prrx2* target genes.

To further evaluate the senescence-associated features of distinct fibroblast subpopulations in the aortas of *Lmna^G609G/G609^* ^G^ mice, we performed SASP scoring. Our analysis revealed a significant increase in the SASP score across seven fibroblast subpopulations in *Lmna^G609G/G609^* ^G^ mice, including Fib1, Fib2, Fib4, Fib5, Fib6, Fib7, and Fib9 (**Figures 6A, B**). These results indicate that various fibroblast subtypes in the progeroid aorta of HGPS mice are universally characterized by chronic inflammation.

To investigate intercellular interactions among fibroblast subpopulations, we analyzed cell-cell communication networks across nine subtypes. We observed a decreased frequency and increased strength of interactions between fibroblast subpopulations in the aortas of HGPS mice (**Figures 6C, D**). In addition, we observed an increase in the frequency and intensity of cell communication in Fib3 and Fib9, and Fib4 (**Figures 6E, F**). These findings suggest that fibroblast subpopulations in HGPS aortic aging are not uniformly functionally suppressed; instead, they remodel the pathological microenvironment through functional specialization and compensatory signal amplification.

Next, we performed a single-cell regulatory network inference and clustering (SCENIC) analysis, which identified the transcription factors *Prrx2*, *Foxd1*, and *Hoxc6* as key regulators of the transcriptome dynamics of aortic fibroblasts in *Lmna^G609G/G609^* ^G^ mice (**Figure 6G**). Transcriptional network analysis predicted that these factors act upstream of distinct genes involved in extracellular matrix organization, vascular endothelial growth factor signaling, and collagen fibril organization (**Figures 6H, I**).

### 2.7 Lgals3bp serves as a critical regulator of inflammation and fibrosis in HGPS fibroblasts

**Figure 7:**
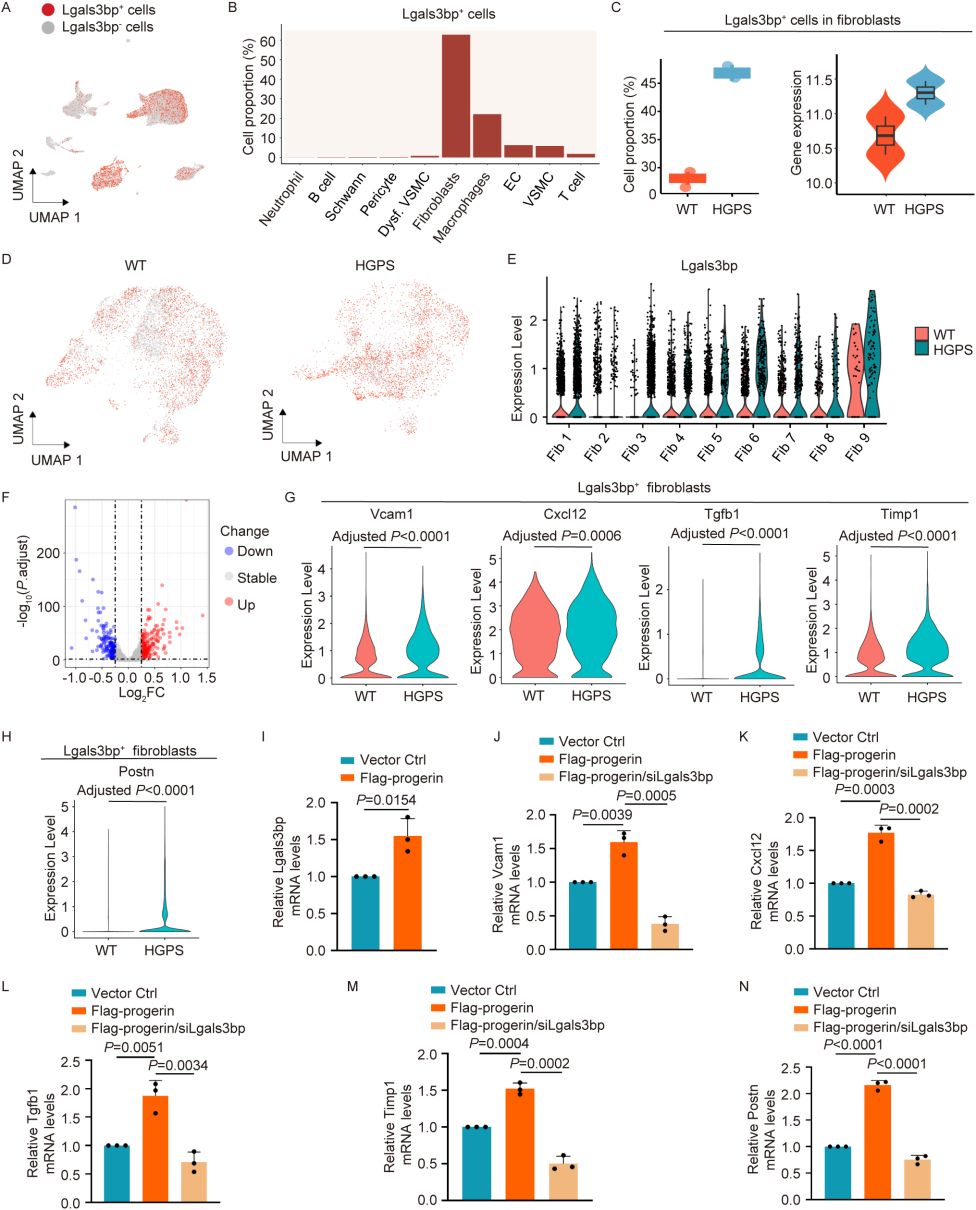
*Lgals3bp* mediates pro-inflammatory and pro-fibrotic functions of fibroblasts in aortic aging. **(A)** UMAP plot showing the distribution of *Lgals3bp^+^* cells in different cell types of the mouse aorta. **(B)** Bar plot showing the proportions of *Lgals3bp^+^* cells across different cell types in mouse aorta. **(C)** Box plots showing the proportion of *Lgals3bp^+^* cells and violin plot showing *Lgals3bp* expression level in WT and HGPS fibroblasts. **(D)** UMAP plots showing the distribution of *Lgals3bp^+^* cells in aortic fibroblasts from WT and HGPS mice. **(E)** Violin plot showing the *Lgals3bp* expression level in different fibroblast subpopulations of WT and HGPS. **(F)** Volcano plot showing DEGs in *Lgals3bp^+^* fibroblasts from WT and HGPS mice. **(G, H)** Violin plots showing the expression levels of *Vcam1*, *Cxcl12*, *Tgfb1*, *Timp1* **(G)**, and *Postn* **(H)** in *Lgals3bp^+^* fibroblasts from WT and HGPS mice. **(I)** qPCR analysis of *Lgal3bp* mRNA levels in NIH3T3 cells transfected with Flag-progerin. **(J-N)** qPCR analysis of *Vcam1* **(J)**, *Cxcl12* **(K)**, *Tgfb1* **(L)**, *Timp1* **(M)**, and *Postn* **(N)** mRNA levels in progerin-expressing NIH3T3 cells transfected with siLgals3bp. Data are presented as mean ± SD. Statistical significance was determined by two-tailed unpaired Student’s t-test.

To identify the potential drivers of fibroblasts during aortic aging in HGPS mice, we analyzed DEGs in aortic fibroblasts from *Lmna^G609G/G609^* ^G^ mice and found that Galectin 3 binding protein (*Lgals3bp*), a secretory glycoprotein, was upregulated in HGPS aortic fibroblasts. Previous studies have demonstrated that *Lgals3bp* activates the TGF-β1 signaling pathway, thereby promoting hepatic fibrosis in mice (14). To investigate whether Lgals3bp contributes to aortic aging in *Lmna^G609G/G609^* ^G^ mice, we analyzed the cell type-specific expression patterns. *Lgals3bp* was expressed in fibroblasts (62.7%), macrophages (22.1%), ECs (6.3%), VSMCs (5.8%), T cells (1.7%), dysfunctional VSMCs (0.8%), B cells (0.1%), pericytes (0.2%), and neutrophils (0.04%) (**Figures 7A, B**). Compared to *Lmna^+/+^* mice, *Lmna^G609G/G609^* ^G^ mice exhibited a higher proportion of *Lgals3bp^+^* fibroblasts and elevated Lgals3bp expression levels (**Figure 7C**), particularly in Fib3 (**Figures 7D, E**). Furthermore, we found that pro-inflammatory factors (*Vcam1* and *Cxcl12*) and fibrosis-related genes (*Tgfb1*, *Timp1*, and *Postn*) were upregulated in *Lgals3bp^+^* fibroblasts from the HGPS mouse aorta compared to those from WT mice (**Figures 7F-H**). These findings suggest that *Lgals3bp* may drive pro-inflammatory and pro-fibrotic activities in aortic fibroblasts during HGPS pathogenesis.

To validate the expression of Lgals3bp in HGPS fibroblasts at the cellular level, we employed a lentiviral system to express Flag-tagged progerin in mouse embryonic fibroblasts NIH3T3, thereby mimicking the pathophysiological features of HGPS fibroblasts. qPCR analysis revealed that Lgals3bp was upregulated in NIH3T3 cells overexpressing progerin (**Figure 7I**). To investigate whether *Lgals3bp* affects the expression of inflammatory factors and activates fibrosis in fibroblasts, we knocked down Lgals3bp in progerin-overexpressing NIH3T3 cells. The results showed that progerin overexpression induced the upregulation of pro-inflammatory factors (*Vcam1* and *Cxcl12*) and fibrosis-related genes (*Tgfb1*, *Timp1*, and *Postn*), whereas *Lgals3bp* knockdown reduced the mRNA levels of these genes (**Figures 7J-N**). These findings indicate that *Lgals3bp* plays a critical role in the regulation of inflammation and fibrosis in HGPS fibroblasts.

## 3 Discussion

Our study confirmed that *Lmna^G609G/G609^* ^G^ mice underwent senescence-associated vascular remodeling in the aorta. Through reanalysis of scRNA-seq data derived from *Lmna^+/+^* and *Lmna^G609G/G609^* ^G^ mouse aortas, we identified fibroblasts as pivotal contributors to chronic inflammation and fibrosis during aortic aging. Further subclassification of fibroblasts into nine transcriptionally distinct subclusters revealed unique molecular signatures, developmental trajectories, and intercellular communication dynamics. Finally, we discovered that *Lgals3bp* was upregulated in the aortic fibroblasts of *Lmna^G609G/G609^* ^G^ mice and played a key role in modulating inflammation and fibrosis in HGPS fibroblasts. In summary, our findings provide critical insights into the cellular and molecular dynamics driving aortic senescence in HGPS mice, highlighting fibroblasts as key coordinators of vascular remodeling during HGPS aortic aging.

HGPS exhibits characteristics of premature or accelerated aging, and its clinical manifestations share similarities with physiological aging, including short stature, atherosclerosis, and osteoporosis (15). The vascular pathology of HGPS overlaps with normal aging in many key features, such as progressive arterial stiffening, calcification, inflammation, and plaque erosion or rupture (16). However, key distinctions include the dramatically accelerated progression of lesions, marked depletion of VSMCs, pronounced adventitial fibrosis, and a distinct lipid risk profile compared to normal aging (17, 18). A single-cell transcriptomic study of arterial aging in primates revealed increased transcriptional noise in aortic adventitial fibroblasts, particularly in pathways related to ECM organization and lipid response (12). Similarly, an scRNA-seq analysis in naturally aged mice not only confirmed increased transcriptional heterogeneity in adventitial fibroblasts but also showed a rise in fibroblast senescence scores with age. This was paralleled by an early emergence of inflammation-associated senescence signatures and an alteration in the communication network between fibroblasts and other cell types (13). Consistent with these observations in natural aging, our study identified significant transcriptomic alterations in aortic fibroblasts of HGPS mice compared to WT controls, especially in inflammatory and ECM organization pathways. Furthermore, aortic fibroblasts in HGPS mice exhibited elevated SASP scores and significant remodeling of their communication networks with other cell types. Collectively, these findings suggest that the impact of fibroblasts on aortic pathology in HGPS mice shares certain similarities with the processes observed in natural aging.

Building on the original research, our analysis has enriched differential gene expression profiling across distinct cell types in the aortas of HGPS and WT mice. By establishing associations between DEGs with aging and arteriosclerosis, we demonstrated that HGPS aortic cells exhibited transcriptional changes that were shared with the progression of both aging and arteriosclerosis (**Figures 3D-F**). Senescent cells reinforce the local inflammatory microenvironment by secreting diverse inflammatory cytokines and chemokines, which recruit additional immune cells to plaque regions. This process affects neighboring cells, perpetuates senescence, and ultimately drives tissue dysfunction (19, 20). Previous studies have documented pro-inflammatory changes in gene expression profiles of both ECs and VSMCs in aged rodents and primates (21–23). Our findings further reveal that fibroblasts play a critical role in mediating chronic inflammation and modulating cellular communication networks within HGPS aortas, highlighting their previously underappreciated contribution to vascular pathology.

The ECM is a critical determinant of health and longevity (24). Ubiquitously present across all tissues and organs, the ECM not only provides essential mechanical scaffolding, but also mediates sophisticated biomechanical and biochemical signaling required for tissue homeostasis, morphogenesis, and cellular differentiation (25, 26). Mammalian aging induces profound alterations in the biosynthesis of the ECM, post-synthetic modifications of ECM components, and modified cell-matrix interactions, collectively driving the development of age-related pathologies (27). Age-associated dysregulation of collagen synthesis in vascular walls, potentially mediated through enhanced TGF-β paracrine signaling (28), contributes to vascular fibrosis and arteriosclerosis (29). Additional hallmarks of increased arterial stiffness include diminished elastin production, elastin degradation and fragmentation, elastocalcinosis, and altered crosslinking of ECM constituents (30, 31). Our study confirmed that aortic fibroblasts of HGPS mice predominantly participate in ECM-related biological processes (**Figure 2F**), with collagen-related pathways showing upregulated activity in fibroblast-to-dysfunctional VSMC communication (**Figure 4G**), indicating that fibroblasts play a crucial role in the ECM remodeling associated with premature aging.

LGALS3BP, alternatively designated as Gal3-BP, 90K, Mac2-BP, or CyCAP, is a secreted multifunctional glycoprotein that has been implicated in modulating pathological processes across infection (32), autoimmunity (33), multi-organ fibrosis (34, 35), and oncogenesis (36, 37). Clinically, elevated plasma LGALS3BP levels are correlated with long-term mortality in coronary artery disease (38). Experimental evidence from hepatocyte-specific *Lgals3bp*-knockin mice revealed exacerbated hepatic fibrosis accompanied by elevated *Tgfb1* levels. Mechanistically, *Lgals3bp* induces substantial upregulation of *Tgf-β*-regulated genes in hepatocellular carcinoma cells, including established *Tgf-β1* targets, including *Serpine1*, *Vcam1*, and *Il6 (* 14). Consistent with these findings, we observed upregulated *Lgals3bp* expression in the aortic fibroblasts of HGPS mice (**Figure 7C**), with cellular validation demonstrating elevated *Lgals3bp* levels in progerin-overexpressing NIH3T3 (**Figure 7I**). In addition, *Lgals3bp* knockdown significantly reduced the expression of pro-inflammatory factors and fibrosis-related genes in HGPS cells (**Figures 7J-N**). Collectively, our results established *Lgals3bp* as a pivotal mediator driving pro-inflammatory and pro-fibrotic functions in aortic fibroblasts of *Lmna^G609G/G609G^* mice. Further investigation is required to determine the function of *Lgals3bp* in HGPS mice, and whether inhibiting *Lgals3bp* can ameliorate aortic progeroid phenotypes.

This study has some limitations. While scRNA-seq has revolutionized our understanding of cellular heterogeneity, its limitation lies in the loss of spatial information during tissue dissociation. Consequently, it fails to reveal the intricate architectural relationships and cell-cell communication niches that are critical to tissue function. This gap can be addressed in future studies by employing spatial transcriptomics, an approach that enables the overlay of rich gene expression data onto precise histological maps, thereby bridging a critical dimension in interpreting complex processes such as aortic aging. Furthermore, we demonstrated that fibroblasts in HGPS aortas exhibit enhanced communication with dysfunctional VSMCs and that *Lgals3bp* promotes the expression of pro-inflammatory and pro-fibrotic factors in fibroblasts at the cellular level. However, whether *Lgals3bp* exacerbates VSMCs dysfunction by augmenting this crosstalk through fibroblast-derived secretory factors remains to be determined. In addition, we restricted our analysis to *Lgals3bp*-induced upregulation of pro-inflammatory and pro-fibrotic factors in fibroblasts. The underlying mechanisms through which *Lgals3bp* modulates these factors will require dedicated investigation in future studies. Finally, our study primarily investigated the role of fibroblasts in aortic aging-associated vascular remodeling solely in HGPS mice, with validation limited to progerin-overexpressing NIH3T3 cells. It will be essential for future studies to determine whether this mechanism is applicable to physiological aging, and its potential role in natural aging remains to be validated in naturally aged mice or human aortic fibroblasts.

## 4 Materials and methods

### 4.1 Animals

All animal experiments were approved by the Animal Ethics Committee of Changchun Sci-Tech University (Approval No. CKARI2024010) and conducted in accordance with the “Laboratory Animal – Guideline for ethical review of animal welfare” (GB/T 35892-2018) and the “Guide for the Care and Use of Laboratory Animals: Eighth Edition”. Progerin ubiquitously expressed *Lmna^G609G/G609G^* mice (ICR background) were previously described (39), with age-matched wild-type *Lmna^+/+^* littermates serving as controls. Mice were maintained under controlled environmental conditions (22 ± 2 °C, 50% humidity) with a 12-hour light/dark cycle, and provided ad libitum access to food and water.

### 4.2 Histological analysis

Mice of specified genotypes at 14 weeks of age were first perfused with PBS, followed by fixation via perfusion with 4% paraformaldehyde (PFA). Aortic tissues were post-fixed in 4% PFA at 4 °C for 3 days and subsequently embedded in paraffin. H&E, VVG, Masson’s trichrome, and immunohistochemical (IHC) staining were performed by Servicebio. Tissue sections were immunostained with anti-p16 (1:200, Abclonal A0262) and anti-p21 (1:1000, Servicebio GB15531) antibodies. Quantification of protein expression was performed by calculating the percentage of positively stained cells across three randomly selected fields per section. Vascular wall thickness was defined as the radial distance from the luminal surface to the external elastic lamina. Medial cell density was quantified by counting nuclear counts per unit area within the medial layer. Elastin fragmentation was assessed by evaluating the continuity of elastin fibers observed in histological sections. Collagen content was quantified as the percentage of Masson’s trichrome-positive areas relative to the total tissue cross-sectional area.

### 4.3 Cell culture

HEK293T and NIH3T3 cells were provided by Northeast Normal University. HEK293T and NIH3T3 cells were cultured in Dulbecco’s Modified Eagle’s Medium (DMEM) supplemented with 10% fetal bovine serum (FBS) and 1% penicillin-streptomycin, and maintained at 37 °C under a humidified 5% CO_2_ atmosphere.

### 4.4 Lentivirus production and transduction

HEK-293 cells were co-transfected with the lentiviral packaging plasmids pMDlg/pRRE (6.5 μg), VSV-G (3.5 μg), pRSV-Rev (2.5 μg), and the core plasmid pCDH-CMV-3×Flag-progerin (16 μg) using the transfection reagent polyethylenimine (8 μl). After 6–8 hours, the culture medium was replaced with a fresh complete medium. The supernatant collected at 48 h and 72 h post-transfection was filtered through a 0.45 μm filter. Viral particles were concentrated by centrifugation at 4000 rpm for 20 minutes. The concentrated virus was used to infect NIH3T3 cells in the presence of polybrene (5 mg/ml).

### 4.5 siRNA transfection

NIH3T3 cells cultured in 6-cm dishes were switched to serum-free DMEM prior to transfection. For transfection mixture preparation, 8 μl of Lipofectamine 2000 transfection reagent (Invitrogen, USA) was added to 250 μl of serum-free DMEM, gently mixed, and incubated at room temperature for 5 minutes. Separately, 25 μl of 20 μM siRNA (Sangon Biotech, China) was diluted in 250 μl serum-free DMEM and mixed. The two solutions were gently mixed and incubated at room temperature for 20 minutes. The resulting complex was added dropwise to the cell culture medium with gentle swirling. After 6–8 hours of incubation, the medium was replaced with fresh complete medium. Cells were subjected to downstream analyses 48 hours post-transfection.

### 4.6 RNA extraction and real-time qPCR analysis

Total RNA was isolated from cells using TRIzol™ Reagent (Cat# ET101, TransGen Biotech) following the manufacturer’s protocol. cDNA synthesis was performed with the TransScript^®^ Uni One-Step gDNA Removal and cDNA Synthesis SuperMix (Cat# AT311, TransGen Biotech). Real-time quantitative polymerase chain reaction (RT-qPCR) was conducted on a StepOnePlus™ Real-Time PCR System (Applied Biosystems, USA) using PerfectStart^®^ Green qPCR SuperMix (Cat# AQ601, TransGen Biotech). β-actin served as the endogenous control. Gene-specific primers (synthesized by Sangon Biotech, Shanghai, China) were designed with the following sequences:

β-actin (mouse)_fwd (5′-CCTCTATGCCAACACAGTGC-3′) and β-actin (mouse)_rev (5′- ACATCTGCTGGAAGGTGGAC-3′);

Lgals3bp (mouse)_fwd (5′-AGGGCTGCGACCTATTCATC-3′) and Lgals3bp (mouse)_rev (5′-TCGGGAGTAAAAGTACCTGAGG-3′);

Vcam1 (mouse)_fwd (5′-CTGGGAAGCTGGAACGAAGT-3′) and Vcam1 (mouse)_rev (5′-GCCAAACACTTGACCGTGAC-3′);

Cxcl12 (mouse)_fwd (5′-TGCATCAGTGACGGTAAACCA-3′) and Cxcl12 (mouse)_rev (5′- CACAGTTTGGAGTGTTGAGGAT-3′);

Tgfb1 (mouse)_fwd (5′-CTGCTGACCCCCACTGATAC-3′) and Tgfb1 (mouse)_rev (5′- GGGCTGATCCCGTTGATTTC-3′);

Timp1 (mouse)_fwd (5′-CGAGACCACCTTATACCAGCG-3′) and Timp1 (mouse)_rev (5′- ATGACTGGGGTGTAGGCGTA-3′);

Postn (mouse)_fwd (5′-TGGTATCAAGGTGCTATCTGCG-3′) and Postn (mouse)_rev (5′- AATGCCCAGCGTGCCATAA-3′).

### 4.7 Raw data processing and dimensionality reduction-driven clustering analysis

The scRNA-seq dataset associated with HGPS murine aortas was obtained from the study by Ana Barettino et al. (6). This comprehensive dataset comprises transcriptomic profiles from aortic tissue samples derived from two biological pairs of *Lmna^G609G/G609G^* mice and their wild-type littermate controls (*Lmna^+/+^*) on a C57BL/6 genetic background.

Raw scRNA-seq data were processed using the Seurat package (v4.4.0) for normalization, quality control filtering, dimensionality reduction, cell clustering, and differential gene expression analysis (40). Rigorous quality thresholds were applied: cells retaining 200-5,000 detected genes, >1,000 UMIs, and <10% mitochondrial gene content. Potential doublets were identified and removed via DoubletFinder (v2.0.3) (41), while ambient RNA contamination was mitigated using DecontX (v1.4.0) (42). Batch effects across samples were corrected through Harmony integration (v1.2.3) (43), with the top 40 Harmony embeddings selected via the ElbowPlot function for subsequent clustering and visualization. Cell clustering was performed across resolution parameters (0.1-1.2) using the FindClusters function, with optimal resolution (0.7) determined by cluster stability analysis via the cluster R package (v0.5.1). Cell identities were annotated based on canonical marker genes reported in literature. Dimensionality reduction and visualization were achieved using the RunUMAP function implementing UMAP. For fibroblast subpopulation analysis, fibroblast clusters were extracted and re-clustered at a resolution of 0.3 following the aforementioned workflow.

### 4.8 Differential gene expression and GO enrichment analysis

Following stringent quality control, a total of 22196 high-quality cells were retained for downstream analyses. Differential gene expression analysis was conducted using the MAST framework (v1.28.0) implemented through the FindAllMarkers function in Seurat (v4.4.0) (44). DEGs were identified using a conservative threshold of Bonferroni-adjusted P < 0.05 combined with absolute log_2_ (fold-change) > 0.25.

GO enrichment analysis was performed using the clusterProfiler R package (v4.14.0) (45), followed by visualization of the results through the ggplot2 R package (v3.4.4; https://github.com/tidyverse/ggplot2), with representative GO terms selectively displayed to highlight biologically significant functional categories.

### 4.9 Analysis of intercellular communication

Cell-cell communication networks between distinct cellular populations were analyzed using CellChat (v2.1.2) (46), with ligand-receptor interactions considered statistically significant at a threshold of P<0.05. Intercellular communication patterns were visualized through the netVisual circle function, quantitatively depicting interaction frequency and communication strength across cell types.

### 4.10 Pseudotime analysis

Cellular differentiation potential was quantified through transcriptional entropy analysis using CytoTRACE (v1.1.0), a computational framework for predicting developmental potency based on gene expression heterogeneity (47). Pseudotemporal trajectory reconstruction was performed within the Monocle 3 (v1.3.7) environment (48).

### 4.11 Transcriptional regulatory network analysis

Transcriptional regulatory network analysis was performed using the pySCENIC workflow (v0.12.1) (49). The mm10 transcription factor (TF) motif database was retrieved through RcisTarget (v1.28.0). Gene regulatory networks (GRNs) were inferred from DEGs using GRNBoost2. The reconstructed transcriptional networks were visualized using Cytoscape (v3.7.2) (50).

### 4.12 Statistical analysis

Statistical analyses were performed using GraphPad Prism (version 8.0.2). Intergroup differences were assessed by two-tailed unpaired Student’s *t*-tests. Quantitative data were expressed as mean ± standard deviation (SD). Statistical significance was defined as *P* < 0.05.

## Data Availability

The original contributions presented in the study are included in the article/**Supplementary Material**. Further inquiries can be directed to the corresponding author.

## Supplementary material

Supplementary Table 1 Differentially expressed genes (DEGs) in the aorta of *Lmna^G609G/G609G^* mice.
